# Domesticating the Drone: The Demilitarisation of Unmanned Aircraft for Civil Markets

**DOI:** 10.1007/s11948-014-9603-3

**Published:** 2014-11-05

**Authors:** Philip Boucher

**Affiliations:** Digital Citizen Security Unit, Institute for the Protection and Security of the Citizen (IPSC), Joint Research Centre (JRC), European Commission, Via Enrico Fermi 2749, 21027 Ispra, Italy

**Keywords:** RPAS, UAV, Drones, Responsible research and innovation, Dual-use

## Abstract

Remotely piloted aviation systems (RPAS) or ‘drones’ are well known for their military applications, but could also be used for a range of non-military applications for state, industrial, commercial and recreational purposes. The technology is advanced and regulatory changes are underway which will allow their use in domestic airspace. As well as the functional and economic benefits of a strong civil RPAS sector, the potential benefits for the military RPAS sector are also widely recognised. Several actors have nurtured this dual-use aspect of civil RPAS development. However, concerns have been raised about the public rejecting the technology because of their association with military applications and potentially controversial applications, for example in policing and border control. In contrast with the enthusiasm for dual-use exhibited throughout the EC consultation process, the strategy for avoiding public rejection devised in its roadmap would downplay the connection between military and non-military RPAS and focus upon less controversial applications such as search and rescue. We reflect upon this contrast in the context of the European agenda of responsible research and innovation. In doing so, we do not rely upon critique of drones per se, in their neither their civil nor military guise, but explore the extent to which current strategies for managing their public acceptability are compatible with a responsible and socially beneficial development of RPAS for civil purposes.

## Introduction

Remotely piloted or unmanned aviation systems (RPAS/UAS^1^), colloquially known as drones, are aerial vehicles that fly without an on-board pilot, as well as the systems that support them to do so. These systems have varying degrees of automation and autonomy, but usually include human remote pilots who control the vehicle from meters, kilometres or continents away. Perhaps the most established and visible applications of RPAS are for military purposes (M-RPAS), including combat and surveillance operations, but many applications have been identified for domestic uses such as environmental monitoring, security, emergency response, surveillance and recreation. In addition to the significant functional and economic benefits of these civil RPAS (C-RPAS), as a dual-use technology they are also expected to support innovation in the M-RPAS sector. The technologies required for C-RPAS operations are ready for market and the principal barriers to development in the sector are regulatory. In response to demand, the European Commission (EC) has published strategies to allow the gradual integration of C-RPAS into normal airspace.

Interest in societal and ethics aspects of RPAS has largely focussed upon military applications, with debate on the ethics of M-RPAS per se (see Billitteri 2010) as well as the ethics of promoting M-RPAS development through dual-use research (see Sparrow 2012). Some scholars have focussed specifically upon C-RPAS development, usually in the context of civil liberties and most often focussing principally upon privacy, surveillance, and militaristic policing (e.g. Gersher 2013; Salter 2014; Hayes et al. 2014; Straub 2014; Urquhart 2013; Finn and Wright 2014; Galliot 2012; Jones 2014). Here, we focus upon the strategies designed to support the development of the C-RPAS sector, in particular the demilitarisation of C-RPAS discourse as a means of managing their public acceptability. We offer a critique of these strategies with reference to three points drawn from responsible research and innovation (RRI).

RRI is a guiding concept for the technology development and management in Europe, and permeates the EC’s €80b 2014–2020 research funding framework, Horizon 2020. It is described as “an inclusive approach to research and innovation (R&I)” and aims to “better align both the process and outcomes of R&I, with the values, needs and expectations of European society.” (European Commission 2014e). However, RRI remains a concept under development; it is subject to interpretive flexibility and methodological debate. Here, we draw three key points from core RRI literature as a means grounding our critique of the strategies for managing public responses to C-RPAS. The first point is about the purpose of innovation. Responsibility goes beyond the processes or outcomes of a development, extending to the substance and transparency of its purposes and motivations (Stilgoe et al. 2013). The second point is about the role of knowledge as a precondition for responsibility. This has several implications, but for our purposes, it means that relevant publics should become co-responsible for development as agents equipped with adequate knowledge about its motives, aims and consequences (European Commission 2013). The third point is that *pushing* a technology without sufficient dialogue at early stages is irresponsible, and can damage development (von Schomberg 2013).

Our central critique of the strategies for managing public acceptance of C-RPAS is that they are designed to minimise widespread recognition of one of the motivations and expected consequences of their development, that is, the expected benefits for the M-RPAS sector. Instead, the strategies seek to emphasise less controversial applications and downplay associations with military drones. In the context of RRI, we present this demilitarisation as an example of technology push (von Schomberg 2013), an irresponsible approach to development whereby acceptance is sought by reducing the scope of debate and, in particular, debate about the full range of motivations for and expected outcomes of development. We also show how the approach may be self-defeating, leading to increased resistance to the technology, with examples of failed attempts to demilitarise C-RPAS discourse by avoiding the use of the word ‘drone’. While it may be possible to argue that the technology per se is irresponsible, the critique here rests solely upon the strategies for gaining public acceptance as articulated in the Roadmap produced by the European RPAS Steering Group (ERSG 2013b). Our recommendation—also based upon RRI—is that the burden of acceptability is shifted from citizens to the technology. That is to say, we should be seeking means of making C-RPAS development acceptable to citizens, rather than seeking ways of making citizens accept C-RPAS development. In pursuing this agenda, we suggest that a more transparent approach to C-RPAS development could ultimately support the emergence of a more robust sector.

The following section introduces RPAS technology, describing the range of their specifications as well as some potential applications. The “C-RPAS Development in Europe” section describes the European strategy for introducing C-RPAS in domestic airspace, from early consultations in 2009 to the roadmap that has remained the key reference point for European policy since its publication in 2013. Section “Recognising the Dual Use of RPAS” highlights how various stakeholders have explicitly recognised and celebrated dual-use aspects of C-RPAS technology, before “Killing Machine to Robot Helper: Demilitarising Visions of RPAS” section describes strategies designed to manage public acceptability of C-RPAS by avoiding militaristic associations of C-RPAS. In the final section, this contrast is considered in the context of RRI.

## The Technology

RPAS refers to a system, extending beyond the unmanned vehicle to include ground stations (where control units and remote pilots are based) and communications infrastructures. Within the broad definition of RPAS lie a diverse range of systems and vehicles. Some differences between these RPAS are immediately apparent, such as the size or weight of vehicles. Other differences are more subtle, such as the medium of communication between the vehicle and the ground station. These technical differences are particularly important in regulations and the logistical management of RPAS, including the definition of the phases of airspace integration.

The distinction between RPAS and other UAS is defined by the extent of autonomy and automation delegated from the pilot to the system. Automation levels range from those that are fully piloted from a remote location to those that are fully automated. There are also several points in-between, with some manoeuvres triggered automatically through autonomous monitoring of conditions. Depending upon system priorities, autonomous manoeuvres may have priority over, or be overridden by, the commands of a remote pilot. The International Civil Aviation Organisation (ICAO) and current EC plans will only permit autonomous manoeuvres to override pilot command in extraordinary circumstances such as communication failure or imminent collision risk. The UAS technologies beyond this definition, featuring greater autonomy, are also quite well developed and, while integration is not currently planned, it could plausibly follow a successful period of development in the RPAS sector.

RPAS vehicles are often organised by their weight, with 150 kg defining the boundary between heavy and light vehicles. In Europe, light vehicles are regulated at a national level while heavy vehicles are regulated at a European level. The 150 kg threshold has been described as arbitrary (Finn and Wright 2014). The difference in regulatory treatment above and below this threshold could fragment development and, ultimately, have a distorting effect upon the market. Two variables are often used to capture the complexity of RPAS operations; the operating altitude and the line of sight (LOS) from the remote pilot station (RPS). Unlike the weight threshold, these are not arbitrary but capture the complexity of the operational requirements for flight. At low-altitudes, up to 150 m (below the level where manned aircraft fly), RPAS are further differentiated by their LOS from the RPS. In the simplest scenario, the vehicle remains in *direct visual LOS* from the RPS, usually within 500 m. Where this is not possible, *extended visual LOS* describes operations where the vehicle is not directly visible to the pilot and a support crew based in a second location is used to maintain visual LOS. Where this is not possible, the situation is described as *beyond visual LOS*, and support technology is required to monitor movement. At high-altitudes, 150 m, RPAS may share airspace with manned aircraft and so safe integration is crucial making the situation more complicated than lower altitude flights. In a *radio LOS,* a direct connection is maintained between the vehicle and the RPS. Where this is not possible, *beyond radio LOS*, more sophisticated communication channels such as satellite are required.

These distinctions are based upon the specifications of the system, and are often used to support the management of the technology in regulatory or logistic contexts. Many discussions about RPAS, however, are less focussed upon the physical specifications of the technology and more upon its applications, as well as the motivations for and consequences of their development. The uses of the technology are also particularly important when evaluating it in terms of ethical principles, social values, and grand visions such as RRI. Applications of RPAS can be organised into four major categories; military; non-military governmental; commercial; and personal/recreational. There are several examples within each category, some already routinely performed, many at a pre-market demonstration stage and others in the category of predictions, promises and imaginaries of varying plausibility. While an exhaustive list would be unwieldy and futile, the following paragraphs present an overview of each category of use with some key examples.

By far the most visible and debated applications of RPAS fall under the military category (M-RPAS), particularly surveillance and combat applications in the War on Terror. M-RPAS can be piloted from an RPS at the other side of the world, distanced from immediate danger, as well as the mechanisms and consequences of their actions. This clearly presents a lower risk to combatants, a key advantage of M-RPAS over manned aircraft. However, some critics have argued that they may contravene the just war principle that an army should not risk reduce risk to their own soldiers by means that increase risk to civilians within enemy territory (Billitteri 2010). Others have suggested that their efficiency makes military operations more attractive, effectively promoting a more aggressive foreign policy, including military action in foreign territories that are not officially defined as warzones, such as Pakistan and Yemen (Sparrow 2009). Counterarguments hold that military drone strikes comply with international law and are justified by the principle of self-defence and are an appropriate response to the characteristics of modern warfare, whereby enemy combatants are not readily distinguishable from civilians (Billitteri 2010). These, and several other ethical and legal arguments for and against M-RPAS, are often debated in policy, academia and the mainstream media (for a European summary, see Dworkin 2013).

The state may also deploy RPAS in non-military, civil operations. These C-RPAS are most often associated with security and surveillance applications, such as policing and border patrol. Other areas where governments have used C-RPAS include forest management, fire services, air sampling, search and rescue, and infrastructural maintenance. The motive is often value for money, with C-RPAS achieving the same (or higher) performance standards as manned aircraft more efficiently and economically. Furthermore, they present options for performing operations in dangerous or uncertain contexts, such as emergency response, with a lower risk to personnel.

Commercial and industrial applications could include internet provision, agricultural monitoring, entertainment, advertisement, security and surveillance, infrastructural maintenance, and delivery services. Some applications offer new capabilities, while others allow safer, more efficient, or ‘sexier’ performance of existing tasks. RPAS are also well suited to so-called ‘dull dirty and dangerous’ where unmanned solutions are preferred. Some such applications have achieved media interest, such as the rather fantastical, headline-grabbing reports about drone delivery services for pizzerias and bookshops. These stories have attracted some mainstream debate about privacy, safety and liability.

Several applications of C-RPAS are outside commercial and state activities, such as recreation, creativity or some other community or individual purpose. Many enthusiasts are organised into substantial communities with websites, forums and meetings.^2^ Many participants appear to have the technical skills to build and modify their own C-RPAS from prefabricated kits or their own original designs, depending upon their level of expertise. These communities are generally supportive to other members new and old, and an ‘open source’ attitude to innovation is adopted, whereby ideas and solutions are shared across open platforms. Some such RPAS applications have community oriented aims, such as education, creativity, empowerment and activism. Despite the legality of personal uses of RPAS often being unclear, there is a growing market for off-the-shelf products for responsible and rogue users alike.

There is substantial crossover amongst these categories of applications and technologies developed and sold for one purpose can be used for another. For example, an RPAS designed to monitor air may be used by the military to identify potential chemical threats to ground troops, by the government to ensure compliance with air quality regulations, by industry to provide information and forecasting services and by individual ‘citizen scientists’ to monitor their industrial neighbours. RPAS base vehicles are highly customisable and can be configured with different payloads to deliver various marketable services for government, commercial, recreational and military purposes. Several niche applications that may not otherwise have attracted sufficient investment to fund their own development are made financially viable by the provision of a core technological base that can be modified for a wide range of mainstream and niche purposes. An illustrative product is Microflown AVISA’s vector sensors, which can detect sounds, locate their source and initialise actions. The devices are promoted for landing support and mid-air anti-collision systems, valuable in many application domains, as well as for target acquisition systems, detecting gunshots and artillery fire and pinpointing their sources as potential targets (Microflown AVISA 2012). It is clear that innovations such as these, as well as greater miniaturisation and economies of scale, will provide benefits across a wide range of RPAS applications for both civil and military purposes.

## C-RPAS Development in Europe

In Europe, C-RPAS above 150 kg are controlled at a European level by the European Aviation Safety Agency (EASA) while those under 150 kg remain under national rules. While some Member States authorise flights in normal airspace, the EC has responded to pressure to support the development of a European C-RPAS market by harmonising rules. This started with a three-part consultation on RPAS from 2009 to 2012. The first stage being a hearing on light unmanned aircraft led by the erstwhile DG Energy and Transport, the second a high-level conference organised by the EC with the European Defence Agency and the third a set of five workshops organised by DG Enterprise and Industry (DG ENTR) and DG Mobility and Transport (DG MOVE) in 2011–2012. Following these consultations, the insights were brought together into a Working Document (European Commission 2012), and the European RPAS Steering Group (ERSG) was established to plan a series of activities that would lead to the initial integration of RPAS in European airspace by 2016. The ERSG roadmap (ERSG 2013b) was delivered in June 2013, setting out several challenges, issues and potential remedial steps. It is not law, but a unified call for action produced by a range of key stakeholders under the guidance of several Commission services, wherein it remains a key document. The following subsections describe these steps in more detail, highlighting the development of ideas raised early in the consultation process to actions recommended in the roadmap, in particular the strategies for managing public acceptance described in its appendix on social issues (ERSG 2013a).

### The Hearing

The hearing (European Commission 2009) gathered 49 European experts from a range of sectors, many of which were government authorities. At the time, interest was not limited to remotely piloted applications but also automated systems, so the term UAS was applied. They identified significant potential for civil applications and anticipated major growth by 2015, particularly in lighter UAS, <150 kg, where costs are lower. The benefits of these UAS were rehearsed in the context of a wide range of economic and effective operations including firefighting, search and rescue, police work, air monitoring, first response, road collision monitoring and ‘persistent, on-demand observation’ allowing sustained surveillance without pausing for crew replacement. They recognised that military applications dominated the UAS sector, and underlined the role of non-military UAS in offsetting the cost of military UAS; “the future military market for unmanned aircraft alone is insufficient to effectively amortise the high costs of development” (European Commission 2009, p. 2).

The cited benefits of UAS development extended to a truly massive, albeit unspecified potential for a wide range of spin-off products and services, comparable with that prompted by space exploration. The main barrier to this development was seen as regulatory. “Once a legal framework exists, a totally new aerial work service supply industry should spout rapidly” (European Commission 2009, p. 10). The barriers were considered surmountable and the hearing report recommended regulations and to expedite the development of the market. For our purposes, it is noteworthy that many of the issues identified during this first hearing had a strong influence in shaping the subsequent consultations and, indeed, the eventual roadmap for supporting a European C-RPAS sector. In particular, it identified three key motives for C-RPAS development that remained throughout the subsequent consultations. These are the functional benefits of C-RPAS themselves, the economic benefits of a European sector, and the benefits to the M-RPAS sector.

### The High-Level Conference

The next part of the consultation period was the high-level conference (European Commission 2010). This gathered representatives of EU institutions, member states and military, industry and aviation sectors including the US Federal Aviation Authority. Echoing the previous hearing, the potential value of the UAS market was reiterated and the need to remove constraints upon the market was underlined. They agreed to set up a group—comprised of representatives of Member States, relevant European and international organisations, user groups and public authorities —to respond to these issues. Reinforcing once again that benefits for M-RPAS were a motive and expected consequence of C-RPAS development, it was agreed that the “high level group will integrate the military representatives in order to ensure that the dual nature of UAS operations shall be addressed from the outset” (European Commission 2010, p. 3).

### The Five Workshops

In the third phase of the consultation, five workshops were held, focusing on industry and markets, airspace integration, safety, societal dimensions and research and development issues. In each, a range of experts and stakeholders were invited to share perspectives, concerns and suggestions for the sector. The fourth, on societal dimensions, is most relevant here. It was held in November 2011 and had four sessions- issues related to the general framework; responsibility, liability and insurance; privacy and data protection; societal impacts, acceptance and ethics. It is clear that the content of these sessions was highly influential in the corresponding sections of the subsequent roadmap.

Most importantly for our purposes, concerns were raised about C-RPAS’ surveillance capabilities and that citizens may be uncomfortable with the RPAS because of their association with military and police applications. With regards solutions, it was suggested that public acceptance could be secured by informing citizens of the advantages of RPAS, that a transparent process should be adopted and an open debate about acceptable uses promoted, and that ‘privacy by design’ approaches may help ensure a ‘societal compliant use’ of C-RPAS. This represented the first serious consideration of public acceptability of C-RPAS, highlighting concerns about potential opposition, particularly in sensitive applications such as policing and surveillance. While the ideas of transparency and open debates about acceptance uses of RPAS appear promising in the context of RRI, the idea of informing citizens of RPAS’ advantages suggests an imbalanced approach to informing debate. Indeed, there is no strategy for informing citizens of the disadvantages of C-RPAS development, nor for promoting debate on unacceptable uses.

### The Working Document

The three stages of this consultation process were brought together in a working document (European Commission 2012). The content of the report closely follows the expert opinions gathered during the consultation. It is at this point that interest was explicitly limited to remotely piloted vehicles and the terminology in reportage changed accordingly from UAS to RPAS. The working document reiterates the potential functional and economic potential of the technology, and the need for coordinated removal of the barriers to market development. The expectations for growth were high and extended well beyond concrete calculations to the prediction that unpredictable markets would emerge. Echoing earlier comparisons with space flight, the reports establishes an analogy in the innovation potential created by the development of the iPad.^3^ The document also referred to the benefits accruing to the military when M-RPAS are used for non-military purposes. These ‘mutualisation’ benefits include financial income generated through the provision of services (operations, training, etc.) and opportunities for military pilots to build experience. Support for the sector was considered urgent because, if delayed, development would shift elsewhere. The USA had announced ambitious plans around the same time and it was felt that Europe should not ‘lag behind’, potentially missing opportunities.

Finally, the working document also considered three societal aspects of RPAS development that were raised during the consultation. The first was the need to state the potential benefits of RPAS, particularly their application in crisis management situations, which provide relatively clear and uncomplicated social benefits. The second concerned issues with responsibility and liability, ensuring operations are adequately monitored and insured. Third, privacy and data protection aspects were considered. The report suggested that no regulatory change would be required at a European level because European law already protects citizens from infringements of privacy^4^ and data protection,^5^ although it supported the introduction of defences against intrusions during the design stages. Finally, the report also expressed concern that public unease around RPAS, resulting from their association with military applications, could cause problems. It suggested that public confidence and support could be raised through transparency, consultation with bodies such as the European Group on Ethics, and clearly defining which applications would be permissible and which would be forbidden. Here we see that the three key motives for, and expected consequences of C-RPAS development remained as economic benefits, functional capabilities, and benefits accruing to the military sector. We also see the concern about public opposition to their use.

### The ERSG Roadmap

Following these consultations, the ERSG was set up to produce a roadmap for the integration of RPAS into European airspace. It was delivered in June 2013, and built upon the momentum of the consultations described in the previous sections, as reflected in much of its content and the way the issues are structured (ERSG 2013b). It identified 104 government non-military (state and public sector) and 98 civil (largely commercial) operations, and stated that 250 RPAS based businesses were working on around 400 products. Most of these were specialist SMEs working on products at a pre-market stage. The roadmap called for regulatory support to overcome the barriers presented by fragmented regulations. In doing so, safety is considered paramount and it is essential that airspace integration would not present any degradation of standards or place new demands upon existing practices in the manned aircraft sector. C-RPAS must be considered equivalent to manned aircraft in terms of air-traffic control and safety requirements. All operators must be certified by a RPAS authority, which would ensure that the appropriate standards are met, and all RPAS pilots would be required to obtain valid licences. The roadmap itself is a proposed schedule for the staggered integration of C-RPAS into normal airspace. The four stages in Table 1, below, refer to a gradually increasing complexity, with higher altitudes, longer lines of sight, more densely populated areas, and greater trans-European harmonisation.

**Table 1:** The integration of C-RPAS into European airspace

2013	Some limited, light C-RPAS flights under strong regulations without harmonisation
2014–2018	Increased harmonisation and daily operations within visual and extended visual LOS, including urban areas; possibly low-altitude operations beyond visual LOS in isolated areas; some operations at higher altitudes in less congested airspace;
2019–2023	Licenced pilots operate in most airspace categories; full integration at low altitudes, regardless of LOS, expanding to more populated areas; public EU flights complying with different sets of national regulations
2024–2028	Operations in most airspace alongside manned aircraft; common rules envisaged for public EU flights; cross border EU flights without special authorisation of excessive administrative burden.

The roadmap also describes several activities to accompany these stages of integration. These are organised into three categories, each with its own dedicated annex to the roadmap; regulations, strategic research and societal impacts. The most relevant for our purposes is the third annex on societal issues, which is organised into three areas. The first, third party liability and insurance, anticipates the possibility of incidents and compensation claims. It is necessary to establish the responsibilities and liabilities of various actors involved in operations and to ensure that each is capable of providing compensation, perhaps through a mandatory third party insurance scheme. The second area is security, responding to the threat of deliberate misuse, perhaps through malicious use of rogue C-RPAS or hijacking legitimate C-RPAS through physical or electronic means. The third area is protection of privacy and personal data. Following the insights gathered during the consultation, the roadmap considers these to be adequately protected by existing European legislation, with Member States responsible for their national implementation. As additional safeguards, the roadmap recommend Member States to consider their current implementations of Directives in the context of a developed C-RPAS sector, and that privacy-by-design methods may help ensure the protection of citizens’ rights. Integrating privacy and data protection measures during the design stage, privacy-by-design could ensure that data is deleted by default, rather than requiring operator action. The roadmap then moves on to consider potential public opposition to C-RPAS, stating that it “is important to modify the vision of “killing machines” they [citizens] have right now due to the actually military-specific utilisation and to some catastrophic movies.” (ERSG 2013b, p. 30). This approach implies that the military framing is unjustified, and that it should be corrected. However, the synergies and crossovers between the two sectors were recognised at several points throughout the hearings and consultations described here. In the following section, we highlight how the connection between M-RPAS and C-RPAS has been identified and nurtured by several key actors. We focus upon those involved in the EC’s consultation process described in the previous sections, although we also illustrate the thoroughgoing acceptance of these connections with reference to military strategy documents and communities of users.

## Recognising the Dual Use of RPAS

As a dual-use technology, the innovation and economies of scale anticipated for C-RPAS will also benefit the M-RPAS sector. As mentioned in previous sections, these synergies were recognised early in the EC consultation process and were encouraged by a wide range of actors. In this section, we explore this recognition in greater detail. First we revisit the C-RPAS consultation period to highlight how dual-use was encouraged throughout the process leading to the publication of the roadmap before exploring narrative and structural support for dual-use in wider settings; military analyses of C-RPAS and recreational RPAS activities. We do not consider these synergies between C- and M-RPAS, nor the recognition of them, as critique in themselves. Rather, we present them as a point of contrast with the strategies for managing public acceptance.

### In EC Consultations on C-RPAS

First, we established in the previous section that the relationship between M-RPAS and C-RPAS was considered from the beginning of the EC consultation period. Even 2 years before this process, a 2007 study commissioned by the EC noted that while the C-RPAS market at that time relied upon the innovation capacity of M-RPAS, a threshold may be reached where the reverse relationship would emerge with the military re-adapting innovations from C-RPAS (Frost and Sullivan 2007). As the consultation gathered pace, confidence in the synergies grew and dual-use became an established driver of C-RPAS development. One of the key challenges identified for the sector in the 2009 hearing was the need to support broad RPAS development by sharing costs across civil, security and defence sectors because “the future military market for unmanned aircraft alone is insufficient to effectively amortise the high costs of development” (European Commission 2009, p. 2). The recognition of RPAS’ dual-use character is also clear during the second part of the consultation period, the high-level conference, which gathered representatives of EU institutions and EU Member States together with military, industry and aviation bodies from around the world (European Commission 2010). There, two points were agreed. First, that a “commonly agreed civil-military regulatory framework should favour dual utilisations of the unmanned systems and the development of cost-efficient solutions by the European industry” (p. 2) and, second, that the “high level group will integrate the military representatives in order to ensure that the dual nature of UAS operations shall be addressed from the outset” (p. 3). In the final working document on C-RPAS before the roadmap, the use of M-RPAS for civil purposes was described as ‘mutualisation’, recognised as an opportunity to improve performance and reduce costs. Performance would be improved though the increased scale and range of missions performed, allowing operators to gain experience and expertise. The cost of M-RPAS programmes would be offset by revenue generated by providing training and also operational services in the civil domains, citing governmental activities such as border surveillance as an example case (European Commission 2012). In this sense, C-RPAS development would not only benefit M-RPAS development through its greater innovation capacity and economies of scale, but also more directly as civil operations can be outsourced to the military.

### In Military Strategy for M-RPAS

It is clear that the EC, and in particular those involved in the consultation, hold the synergies with M-RPAS as one of its key motives for, and expected outcomes of C-RPAS development. However, we can also point to broader evidence, from outside the RPAS consultation, of benefits to M-RPAS being an expected outcome of C-RPAS development. International security scholar Clouet noted that, just as the domestic market for personal weapons in the USA has brought many advantages to its military strength by reducing the costs of gun development and production, a large market for C-RPAS would benefit the M-RPAS sector. The difference is that the benefits that may be accrued for M-RPAS do not come from a large domestic market for *weaponry*, but for *electronics*, such as sensing devices. This ‘shuffles the cards’, presenting an opportunity for some nations that have fallen behind in global arms races to strengthen their global military position (Clouet 2012). This line of thinking is echoed in several documents of military strategies for the future of M-RPAS. For example, at a thematic meeting on defence, the European Council welcomed European C-RPAS development and highlighted the importance of dual-use research to enhance military capabilities and strengthen the defence industry (European Council 2013). Documents from the UK’s Ministry of Defence were more specific, recognising the strategic importance of C-RPAS development for the ‘future battlespace’: “UAS offer an opportunity to capitalise on commercial sector developments and offer rapid technology insertion. Breakthroughs in the civil sector could be rapidly transferred to military use” (Ministry of Defence 2011, 6–12). They also recognise the economic reality of a strong commercial sector which not only breeds innovations that are useful to the military sector, but may actually be the main source of technological development for the military, such that the future development of M-RPAS rests upon commercial development:

“The changes in world economies over the last 2 decades mean that the military sector is now dwarfed by the economic size and power of the commercial sector. Except perhaps for space, new developments in military systems are therefore likely to come from specialised development of commercial systems rather than vice versa. It is to the commercial sector that we must look for the delivery of future disruptive technology.” (Ministry of Defence 2011, 6–13)

### Amongst C-RPAS Enthusiasts

Dual-use aspects of RPAS have also been recognised and given both narrative and structure support amongst some communities of RPAS enthusiasts. In an example from a US public hearing, a respondent expressed his appetite for domestic drones for self-defence: “[W]e have the entire debate flipped. What we should be talking about is how promptly and quickly the FAA is going to move to recognize the self defense rights of all Americans to deploy drones in many different varieties in order to provide self defense” (FAA 2013). The development of a weaponised personal RPAS sector appears unlikely, and is not aligned with the recreational, community-based, and small-scale commercial interests of most C-RPAS enthusiasts. Singer, for example, paints domestic security options in a different light, pointing out that “one man’s hobby may be another man’s plot” (2012). However, the military ‘fetish’ of C-RPAS development is not only for rogue individuals. Here, we conclude our discussion of how C-RPAS developments’ benefits to M-RPAS are widely recognised by showing narrative and structural support for dual-use in some mainstream recreational C-RPAS activities.

The International Aerial Robotics Competition (IARC) has been running for several years, offering prizes to teams that complete missions designed to encourage innovation in the sector. The most recent competition involved navigating an obstacle course and retrieving an object under a set of predefined conditions (see IARC 2013). It had a strong military framing, with the scenario designed as a counterterrorism operation (see video; IARC 2014). The competition rules were described in the form of a top-secret mission from the ‘the Chief of the Bureau of Covert Actions’ which would be set in a ‘security compound near Rafq in the Hesamic Republic of Nari’. Competitors were required to design a vehicle that would autonomously enter a pseudo Islamic terror cell’s compound without detection, either avoiding or destroying its surveillance systems. The ability to read Arabic would support its self-navigation to an office where it would retrieve a USB storage device and replace it with a dummy before escaping the compound. The scenario was presumably designed to ‘spice up’ an otherwise rather dry technical exercise but, given the plethora of metaphors that could have been deployed for the same set of RPAS manoeuvres, the military theme remains an interesting selection and is an illustrative example of the popularity of militarist narratives amongst some RPAS enthusiast organisations.

A second C-RPAS competition, UAVForge, did not exhibit the same narrative militarism but was explicitly designed to capitalise upon dual-use aspects of C-RPAS to support M-RPAS development. Since 1958, the Defense Advanced Research Projects Agency (DARPA) has been the primary innovation engine of the US military, with a mission to maintain the technological superiority of the US military and a ring fenced $2.8b budget (Shachtman 2012). In 2011–2012, along with Space and Naval Warfare Systems Center Atlantic (SSC Atlantic, a US intelligence agency within the Navy), they organised and hosted the UAVForge competition to harness citizen-led innovation to produce military grade equipment and promote innovation in the military UAV sector. A DARPA representative described the aims of the competition; “a capability of military relevance … We seek to lower the threshold to entry for hobbyists and citizen scientists, hoping to yield greater innovation, shorter timelines, better performance and more affordable solutions.” (Federal News Service 2011). They provided a virtual environment for international collaboration in designing and building UAVs and offered $100k, a manufacturing subcontract and an ‘exclusive operational military demonstration’ to the winners. The narrative militarism of IARC was absent, but the operation was designed with M-RPAS in mind. For example, the UAV had to be contained in a ‘soldier portable’ rucksack, it had to find and navigate to an identified observation point without being detected by surveillance systems and ‘perch’ there, sending real-time images back to the operator (DARPA 2012).

These points demonstrate the recognition of the relationship between military and C-RPAS development amongst a wide range of actors. Revisiting the ESRG (2013) roadmap strategy for managing public acceptance of RPAS, this strong link between C-RPAS and M-RPAS is also assumed to be held by citizens in their visions of ‘killing machines’. At this point, however, the relationship is no longer celebrated in the way described so far, but is considered a point of concern. The strategy refers to the need to modify citizens’ visions of C-RPAS to ensure their acceptance. Industry representatives and C-RPAS enthusiasts have also sought to distance the technology from M-RPAS in public-facing material. In the following section, we explore these strategies for the demilitarisation of C-RPAS discourse.

## Killing Machine to Robot Helper: Demilitarising Visions of RPAS

Visions of a technology include how the technology is framed (for example as a solution or a threat to something), and the central metaphors that are used to refer to or explain their role. As discussed, the ERSG roadmap (ERSG 2013a) highlighted concerns about potential public opposition to C-RPAS. It argued that citizens hold a *Killing Machine* vision of RPAS that stems from military use and media depictions of the technology, and that this should be changed (p. 30). They also elucidate a strategy of promoting alternative visions. This strategy is built upon aspects of C-RPAS development that citizens would recognise as beneficial, and has two stages. First, to identify the aspects of RPAS development that are most widely recognised as beneficial and familiar to citizens and, second, to use this information in ‘coordinated actions’. This is distilled into a specific recommendation that captures the simplicity of the approach:

“Description of the issue: The RPAS are actually not known or just known as “killing machines.

Proposed solution: Give to the citizens a different vision” (p. 36)

Alternative visions of C-RPAS, to replace that of the ‘killing machine’, are not articulated, although we may draw some inspiration from the two other motives and expected consequences of C-RPAS development (aside, that is, from their benefits to the M-RPAS sector). These are their functional benefits and economic potential. The first can be captured as a *Robot Helper* vision of C-RPAS as assistants in the tasks that are too dull, dirty, dangerous, difficult or expensive for human presence. Here, RPAS are the enablers of services that deliver social value more effectively and at a lower cost. The second can be captured in a *New iPod* vision of C-RPAS as a revolutionary technology that will spearhead innovation and the inception of new services in other sectors. These predictions go beyond direct, specific advantages—such as improvements in communications or aviation technologies—extending to further unspecified spin-offs on a scale described as *beyond prediction* through comparisons with other technological milestones including space flight (European Commission 2009), the iPod (European Commission 2012) and the internet (European Commission 2014d). These New iPod and Robot Helper visions are quite compatible and are often deployed together, for example in a recent Communication from the EC to the European Parliament and the Council (European Commission 2014a).

While the roadmap does not develop detailed plans for replacing the killing machine vision of C-RPAS, it does introduce some strategies for public communications that, they anticipate, could reduce the possibility of an adverse public reaction. At this point, the roadmap shifts from its previous focus on the different technical specifications of C-RPAS to their different possible uses, arguing that “it is likely that the public will make a distinction between those operations that provide a “greater good” (for example fire fighting or search and rescue) and those operations, which have only limited community benefits” (p. 39). The strategy is further elaborated, underlining the need for better explanation of the potential *benefits* of C-RPAS by highlighting some uses that are considered more benign while avoiding discussion of potentially controversial uses. For example, they recommend the C-RPAS community to “stress the roles RPAS have in conducting humanitarian operations or in testing for airborne toxins, rather than focusing only on the military and security applications” (pp. 39–40). Finally, it recommends establishing an ad hoc RPAS public acceptance facilitator group, which would be tasked with understanding public perspectives on C-RPAS, conducting short surveys, and identifying the means to gain their acceptance.

Strategies for managing visions of C-RPAS may include strategies to manage the vocabulary associated with it, such as refraining from using the word ‘drone’ because it is considered to be too strongly associated with controversial military operations and that this association would damage the C-RPAS sector. In some quarters, the ‘d-word’ (as coined by Gosztola 2013) has become taboo (Grossman 2013), and its use is even disputed in military contexts (see Hammond 2013). Communities of enthusiasts appear to recognise the role of vocabulary in broader visions of technology, and have debated strategies of how to refer to C-RPAS in this context.^6^

Many actors have attempted to manage the vocabulary, often with disappointing results. The Association for Unmanned Vehicle Systems International (AUVSI), which lobbies on behalf of both M-RPAS and C-RPAS sectors, has organised a workshop titled *Privacy, Ethics, Perception and Dealing with the* “*D*” *Word* (AUVSI 2014). Their president argued that the “average person on the street, and even intelligent and informed people, when they think of the word ‘drone,’ they think of the military, they think hostile, they think weaponised, they think large and they think autonomous” (Whittle 2013). Perhaps unsurprisingly, these efforts have often failed, and have collectively yielded several articles about the ‘drone industry that dare not speak its name’. There are several specific examples of attempts to convince journalists to avoid using the word ‘drone’ backfiring. For example, a CEO’s pleas for a journalist to avoid the dreaded nomenclature being cited in the article’s opening line—“whatever you write, please don’t call it a drone” (Terris 2013). These episodes illustrate how difficult it is to control language, particularly in the presence of media interest. When proponents are sensitive to the connection between C-RPAS and M-RPAS, they attract attention to the very thing they wanted to disguise and, perhaps more damaging, they attract attention to the fact that they attempted to disguise it.

The EC have mostly referred to RPAS and UAS in their consultation documents and ‘civil drones’ in public-facing communications (e.g. European Commission 2014b, d, f). Some other policymakers, however, are also considering the role of vocabulary as an important part of the wider vision that is communicated to publics. For example, a UK parliamentarian stated that “using the term drone on smaller UAVs muddied the debate” and “invoked images of the large scale UAVs operated by the military” (All Party Parliamentary Group on Drones 2013), and a UK Defence Committee consultation on military and civil aspects of RPAS development positioned nomenclature at the top of the agenda; “defining the terms RPAS, UAS and “drone”” (Defence Committee 2013).

## The Demilitarisation of C-RPAS: Irresponsible Innovation?

The propriety of M-RPAS and the ethics of dual-use technology development are important topics, but discussion of these issues is not necessary to show that the strategies for managing public acceptance of C-RPAS, as described in the roadmap, do not conform to the concept of RRI. The strength of the relationship between C- and M-RPAS is recognised by stakeholders across the board, with the benefits for M-RPAS consistently identified by policy actors as one of the motives for, and predicted consequences of, the development of C-RPAS. However this contrasts with the visions of and motives for C-RPAS development as presented in the strategies for managing their public acceptance articulated in the ERSG roadmap. As such, the strategies appear disingenuous, as they accept that citizens’ perspectives on technologies are important, but decline to present a full understanding of all the motivations for and consequences of development, most notably that a developed C-RPAS sector would benefit the M-RPAS sector. This problem can be captured in the context of RRI.

Applying RRI to specific research and innovation practices is not straightforward, although guiding frameworks are emerging from academic and policy circles. In the introduction, we outlined three key points from this literature. These are explored in more detail here with reference to the problematic strategies for managing public acceptance of C-RPAS. The first point was that responsibility goes beyond the processes or outcomes of a development, extending to the substance and transparency of its purposes and motivations. In developing a framework for applying RRI to specific projects, Stilgoe et al. (2013) described moving beyond questions about the products and even the processes of innovation, to interrogate the purposes. This means that, in addition to the direct outputs of an innovation process (the product) and the procedures associated with its management (the process), we must also consider the motivations for an innovation process (the purpose) and their transparency. Proper application of their ‘stage-gate’ method of assessing projects would require the cooperation of agents in control of C-RPAS development with an independent assessment panel. Nonetheless,  we can consider two of their criteria as particularly relevant here; *clear communication of the nature and purpose of the project*, and *mechanisms identified to understand public and stakeholder views* (Stilgoe et al. 2013, p. 8). These require the assumptions, commitments and framings of the project to be reflected upon and communicated in dialogic engagement with a wide range of stakeholders and publics, a process that has certainly not occurred during the consultation processes described here.

The second point was about the role of knowledge as a precondition for responsibility. A recent report (European Commission 2013) proposed that if the consequences of an action are not known—perhaps because of unpredictability or misinformation—then the actor cannot be held accountable for them. This ‘criteria excuse’ does not apply to deliberate ignorance or professional negligence, but it does mean that responsible innovations must be designed and managed in “such a way that it provides the relevant persons and stakeholders with relevant knowledge” (p. 57). While the strategies for managing public acceptance of C-RPAS described here have recognised the importance of public awareness and support, the approach has not been to present a complete and balanced understanding.

The third point was that pushing a technology without sufficient dialogue at early stages is irresponsible, and can damage development. In identifying types of irresponsible research and innovation, von Schomberg (2013) described *technology push* with the example of genetically modified (GM) food in the 1990s. Echoing the current discussion of C-RPAS, the irresponsible dimension he identified was not the GM technology itself, but insufficient dialogue amongst actors at an early stage and a regulatory focus upon safety aspects without considering the broader contexts, including social aspects. RRI requires greater stakeholder involvement at earlier stages of development. The innovator must respond to societal needs, which must be defined by societal actors. Such an approach requires the innovator and the societal actors to be co-responsible for the innovation process.

Considering the story of C-RPAS development to date in the context of these warnings, we can point to some specific problems as well as potential remedial action. The EC’s C-RPAS consultation process, described in the “C-RPAS Development in Europe” section, is well documented in a central portal which is regularly updated and includes details including the minutes of meetings and even the slides used during the presentations (European Commission 2014c). Contributions offered to the process were clearly influential in the structure and content of the eventual roadmap, but were limited to expert communities, largely from relevant industry and policy communities with some limited academic involvement. The use of a range of experts and documentation of the process is laudable, but it also exposes the notable absence of public consultation. A second point is that the recommendations for managing public acceptance of C-RPAS rest upon several untested assumptions, including that RPAS are seen as ‘killing machines’, that this is because of media hype, and that this will negatively affect acceptance of C-RPAS. In fact, we know very little about public reactions to C-RPAS as there have been no public consultations. The relatively small number of studies performed to date (in North America) indicate that citizens hold sophisticated understandings of C-RPAS that are not well captured as opposition or support but, rather, depend upon the specific application area and context of use (Bracken-Roche et al. 2014; Eyerman et al. 2013). While the ERSG strategy suggests studies of public attitudes, their aim would be to support the design of better PR campaigns to achieve acceptance more effectively, treating citizens’ objections as obstacles to be overcome (Hayes et al. 2014).

Responding to these points, we recommend a different approach to managing public acceptance of C-RPAS. At present, the burden of acceptability is with the citizen, the target of information actions that are designed to transform them from the passive role of opponent to the passive role of acceptor. In a responsible process, the citizen’s role would be that of a fully informed, active agent, (co-)responsible for development, and the burden of acceptability should be with the technology. As such, strategies to manage public acceptance should seek to transform the technology so that it is acceptable to citizens, not to transform publics into acceptors of the technology. Attempts to replace the killing machines vision with a more palatable alternative may be misguided, if not irresponsible. Following the lessons of those that sought to avoid the use of the word ‘drone’, it may also increase the chance of public opposition. Instead of changing visions, at this early stage of development, a responsible strategy would seek to promote transparent debate and dialogue about a wide range of visions and explore how publics can become part of the co-production of new visions and responsible development paths.

## Footnotes

1
*RPAS* is the term de rigueur in Europe, while *UAS* is more common in the USA and *drone* dominates media and colloquial use.

2 DIY Drones (www.diydrones.com), for example, described as ‘the leading community for personal UAVs’.

3 “The current market for commercial RPAS services is practically non-existent … It is expected that once the barriers limiting RPAS flight will be removed the understanding of the RPAS potential will quickly spread amongst potential users creating new markets of aerial services, in the same way that iPad created an entirely new and unpredicted market for mobile data services.” (European Commission 2012).

4 In the Charter for Fundamental Rights, which enshrines the right to private life and family life.

5 In Directive 95/46/EC and its 2009 revision, which differentiated between commercial and state use of data.

6 “Seriously…..QUIT calling them Drones! They are RC QuadCopters! As an aviation photographer, that word (thanks to the media) has turned a pretty awesome gadget thats been around for years, into another one of their Freak Everyone Out Conspiracies” (Commenter #1 on Couts 2014). “the more we can use it in normal, non-threatening contexts, the less poignant the “scary drone!” talking point will be. Plus, UAV, UAS, and quadcopter all lack the so-called branding power that drone has. I am simply doing what I can to re-brand” (Commenter #2 on Couts 2014).
